# Case Report: Two neonatal cases of genetically confirmed junctional epidermolysis bullosa in a tertiary care center

**DOI:** 10.3389/fped.2026.1785578

**Published:** 2026-03-23

**Authors:** Mohamad Hammoud, Soundos Youssef, Dana Maria Khoury, Malak Jbahi, Ali Dghaily, Salah Yamout, Rama Bdeir, Sara Mchad, Mazen Kurban, Samir Akel

**Affiliations:** 1 Department of Pediatrics and Neonatology, Centre Hospitalier Universitaire d'Orléans, Orléans, France; 2 Department of Pediatrics and Neonatology, American University of Beirut Medical Center, Beirut, Lebanon; 3 Department of Dermatology, American University of Beirut Medical Center, Beirut, Lebanon; 4 Department of Surgery, American University of Beirut Medical Center, Beirut, Lebanon

**Keywords:** Carmi syndrome, dystrophic EB, EB-PA, epidermolysis bullosa, junctional, Kindler epidermolysis bullosa, pyloric stenosis

## Abstract

**Background:**

Epidermolysis bullosa (EB) comprises a group of genetically heterogeneous disorders caused by defects in proteins responsible for dermoepidermal adhesion. Severe forms frequently present in the neonatal period and may be associated with early extracutaneous involvement, reflecting the widespread expression of these structural proteins beyond the skin.

**Case presentation:**

We report two term neonates presenting at birth with extensive mucocutaneous blistering. The first neonate demonstrated congenital skin fragility associated with radiologic evidence of pyloric atresia. Surgical correction was performed, and genetic testing was conducted to clarify the underlying subtype. The second neonate developed rapidly progressive blistering with nail involvement, mucosal lesions, and electrolyte disturbance. A molecular analysis by whole-exome sequencing identified biallelic pathogenic truncating variants in LAMB3 (c.3043C>T [p.Gln1015*] and c.3247C>T [p.Gln1083*]), confirming autosomal-recessive junctional EB. Early genetic evaluation allowed for precise subtype classification and informed multidisciplinary management and family counseling.

**Discussion:**

These cases highlight several important aspects of neonatal care. Early extracutaneous manifestations, particularly gastrointestinal and urogenital involvement, should raise suspicion for integrin- and laminin-related EB subtypes. Prompt genetic testing is essential in the neonatal period to distinguish between clinically overlapping EB phenotypes, refine the prognosis, and guide anticipatory management. Supportive care requires significant modification to routine neonatal practices, including atraumatic handling, specialized wound and mucosal care, nutritional optimization, and close surveillance for systemic complications. The identified mutations further contribute to emerging genotype–phenotype correlations in severe EB.

**Conclusion:**

Severe EB presenting at birth represents a life-threatening multisystem disorder rather than an isolated skin disease. Early genetic diagnosis is essential for accurate classification, prognosis, and family counseling. Detailed neonatal case reports with molecular characterization contribute to improved clinical recognition and understanding of genotype–phenotype correlations in EB.

## Introduction

Epidermolysis bullosa (EB) is a cluster of rare genetic diseases with a heavy burden and no definitive cure to date. It is characterized by tissue fragility secondary to minor mechanical friction or trauma, leading to blisters, erosions, and ulceration (1). More than 30 EB subtypes have been identified and are broadly categorized according to the level of tissue cleavage within the dermoepidermal junction into EB simplex, junctional EB (JEB), dystrophic EB, and Kindler syndrome (1).

Some patients present with isolated skin findings, while others have severe forms of EB that frequently extend beyond cutaneous manifestations and involve epithelial-lined organs such as the gastrointestinal, respiratory, and genitourinary tracts, reflecting the widespread expression of structural adhesion proteins, including integrins, laminins, and collagens (1, 2). In addition, there are two rare forms of EB linked to plectin gene (PLEC1) mutations: one associated with congenital pyloric atresia (EB-PA) and another with late-onset muscular dystrophy (EB-MD) (2).

Furthermore, the EB genotype appears to be associated with phenotype, as the time of presentation and prognosis are quite variable. On one end of the spectrum, there is early infancy fatality in spite of surgical repair of pyloric atresia, and on the other, there is late-onset skin lesion eruption, taking months to years before establishing a diagnosis of EB (3–5). Therefore, antenatal screening for EB using ultrasound and genetic analysis is extremely crucial. Indeed, determining the EB subtype plays a prognostic role and constitutes a pivotal tool to offer genetic counseling to families.

Prenatal testing has become feasible through fetal DNA analysis if fingerprint mutations are known or fetal skin biopsy analysis using transmission electron microscopy and indirect immunofluorescence mapping (6, 7). Moreover, a skin biopsy could be an option to diagnose EB. Nonetheless, its diagnostic value remains uncertain as it is an invasive procedure, especially in the setting of frail skin in EB, and requires repeat biopsies due to inconclusive results (8). Previously, EB diagnosis relied on immunofluorescence mapping and transmission electron microscopy, followed by Sanger sequencing of candidate genes (9). The advent of next-generation sequencing (NGS) has markedly improved diagnostic accuracy and efficiency, enabling comprehensive and rapid identification of pathogenic variants across EB-associated genes (10–14). Early molecular confirmation is essential for precise subtype classification, prognostic stratification, and genetic counseling.

Available therapies mainly provide symptomatic relief. Postnatal treatment is mainly based on novel genetic studies (15), focused on transgenic stem cells (16), transplantation of genetically modified epidermal stem cells (17), recombinant adeno-associated virus-mediated homologous recombination (18), or COL7A1 gene replacement for dystrophic EB (19). The phenotype of EB lies on a spectrum, ranging from mild to lethal, with the mortality rate during infancy and childhood being greatest in junctional EB (JEB), with cumulative and conditional risks of 40%–44.7% by age 1 (20). Severe cases can be disabling due to the profound morbidity and mortality risks of epithelial-lined multiorgan involvement, scarring, and aggressive skin neoplasms (1).

Herein, we report two neonates presenting at birth with severe EB, in whom whole-exome sequencing identified pathogenic variants in ITGB4 and LAMB3. These cases highlight genotype–phenotype correlations and the complexity of neonatal management in severe JEB.

## Case report

The first case relates to a male neonate who was delivered at 39 + 1 weeks of gestation via elective cesarean section to a 29-year-old G2P2 mother. The pregnancy was uneventful, except for mild third-trimester polyhydramnios. There was no history of parental consanguinity. Routine antenatal investigations, including non-invasive prenatal testing, were normal. The birth weight was 3,150 g, length was 50 cm, and head circumference was 34.5 cm. The Apgar scores were 8 and 9 at 1 and 5 min, respectively.

The mother reported a previous term infant who developed blistering skin lesions shortly after birth and died at 48 h of life secondary to severe dehydration. No diagnostic workup or autopsy was performed, and medical records were unavailable.

**Figure 1:**
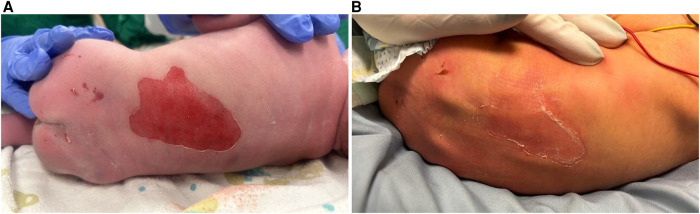
**(A)** A large erosion over the back, present at birth. **(B)** Healing erosion at 1 week of life.

At birth, the neonate exhibited a 3 × 4 cm denuded erosive plaque over the lumbosacral region (Figure 1A), two small tense bullae over the umbilical stump, and a solitary intact bulla over the right wrist. No generalized skin involvement was observed. The scalp and face were spared.

A comprehensive mucocutaneous examination revealed intact oral and nasal mucosa, normal conjunctiva, and no genital or perianal erosions. Nails were normal. No dysmorphic features were identified.

**Figure 2:**
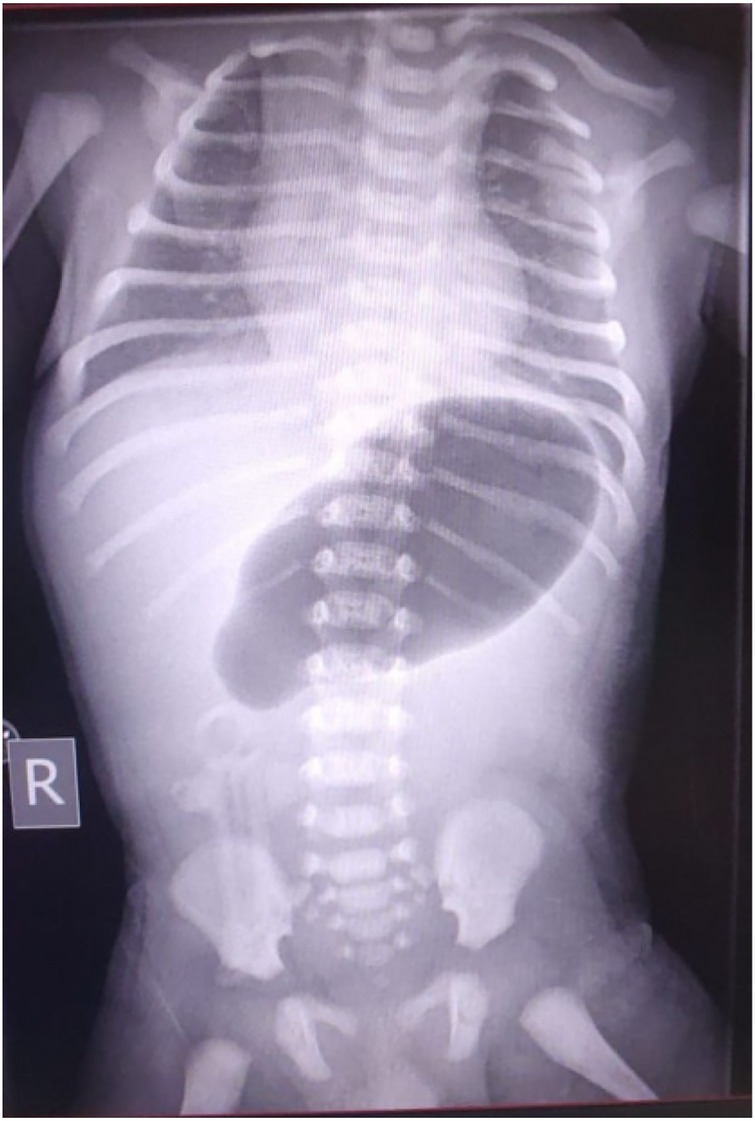
An Abdominal x-ray showing a single bubble sign consistent with pyloric atresia.

An abdominal examination revealed mild distension. An abdominal radiography demonstrated a distended stomach with the absence of distal bowel gas (single bubble sign), highly suggestive of pyloric atresia (Figure 2). Echocardiography and abdominal-pelvic ultrasonography revealed no additional anomalies.

Given the association between neonatal blistering and pyloric atresia, inherited EB, particularly EB-PA, was strongly suspected. Differential diagnoses included staphylococcal scalded skin syndrome, bullous impetigo, congenital herpes infection, incontinentia pigmenti, and dystrophic EB. Infectious evaluation was negative, and clinical findings supported inherited EB.

The parents elected to provide supportive and palliative care since the diagnosis of severe EB was made. Whole-exome sequencing (WES) for both the baby and the parents was performed. WES was selected over a targeted EB gene panel because of institutional availability, rapid accessibility in critically ill neonates, and the ability to simultaneously assess a broad range of genes implicated in inherited blistering disorders.

Whole-exome sequencing revealed a heterozygous, likely pathogenic, variant in ITGB4 [NM_000213.5: c.3451C>T, p.(Arg1151Cys)], classified according to ACMG Class II, consistent with epidermolysis bullosa with pyloric atresia (EB-PA). This variant was predicted to be deleterious by multiple in silico tools. ITGB4 encodes integrin β4, a critical hemidesmosomal protein required for dermoepidermal adhesion. In the appropriate clinical context, these findings support the diagnosis of epidermolysis bullosa with pyloric atresia.

Initial management consisted of nil per os status, total parenteral nutrition via a peripherally inserted central catheter, and meticulous atraumatic wound care using non-adhesive dressings. The handling protocols minimized the friction and shear forces. During the first week of life, the cutaneous lesions demonstrated progressive re-epithelialization without rapid disease progression (Figure 1B).

**Figure 3:**
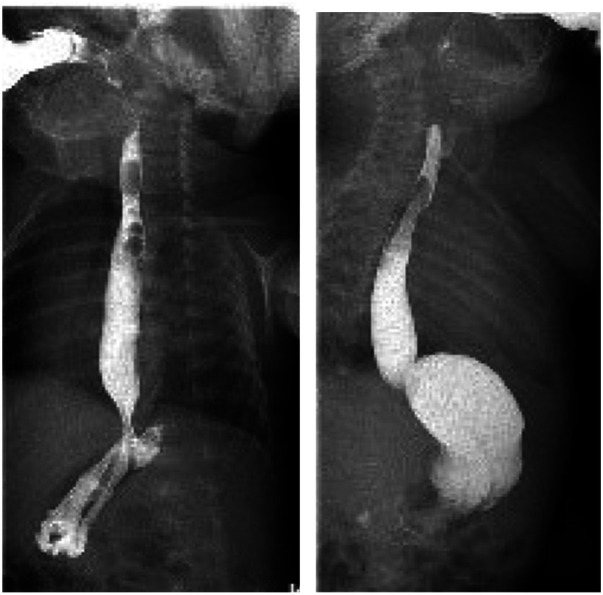
A fluoroscopic swallow postoperatively showing the passage of contrast.

**Figure 4:**
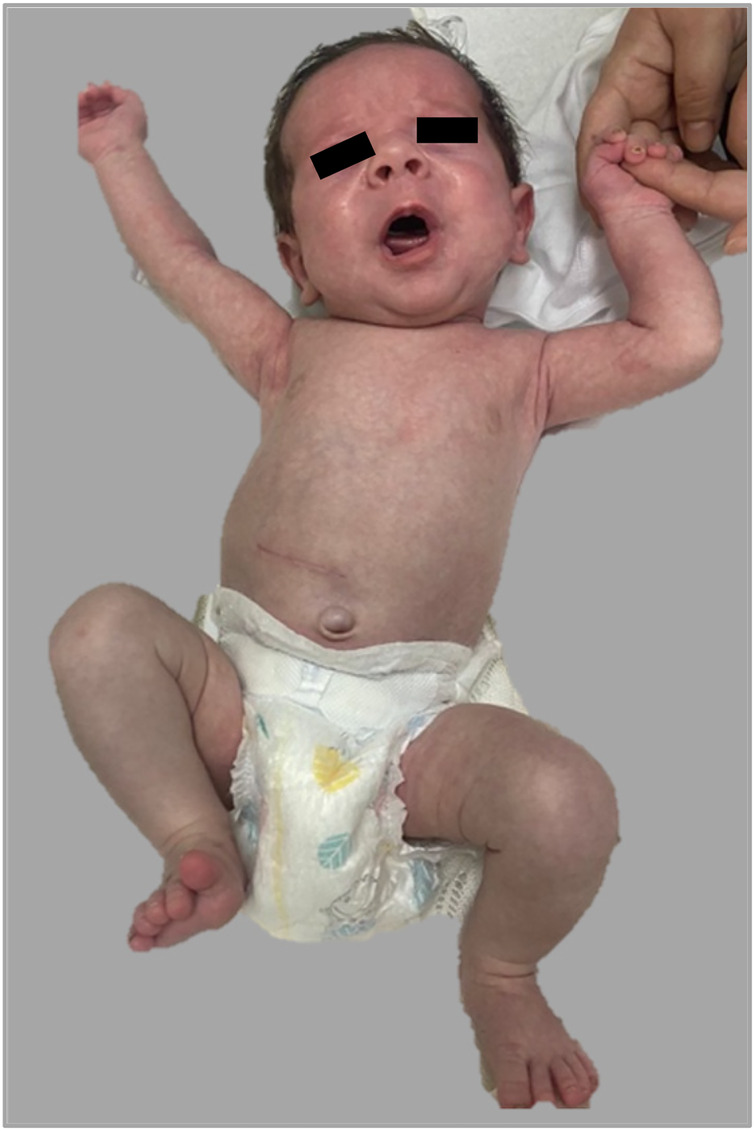
At the time of discharge, with intact skin.

Over this period, disease progression was noted to be slow and not as severe as initially predicted. Following a multidisciplinary reassessment involving neonatology, dermatology, pediatric surgery, and palliative care teams, pyloroplasty was performed on the 10th day of life without complications. Postoperative management included intravenous antibiotics for 7 days and proton pump inhibitors for gastric protection therapy. An upper gastrointestinal contrast study confirmed pyloric patency (Figure 3), and enteral feeding was gradually introduced. Full enteral nutrition was achieved by week three. Throughout hospitalization, skin lesions progressively improved with minimal new blister formation. The infant regained birth weight and tolerated full oral feeding. He was discharged on the 36th day of life with a scheduled dermatology follow-up (Figure 4). Long-term outcomes beyond discharge were not available at the time of submission.

In the second case, a male neonate was born at 38 + 5 weeks of gestation via spontaneous vaginal delivery after an uncomplicated pregnancy. There was no parental consanguinity or a family history of blistering disorders.

The birth weight was 2,980 g. The Apgar scores were 9 and 9 at 1 and 5 min, respectively.

**Figure 5:**
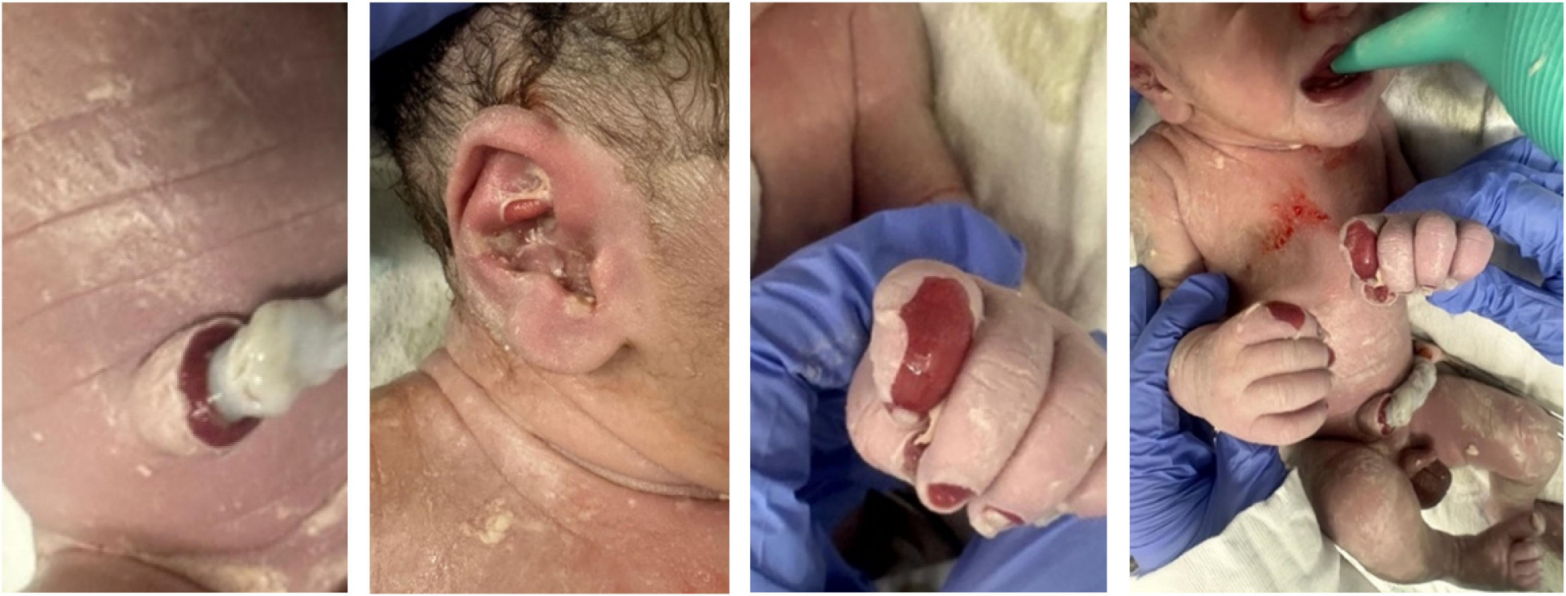
Scattered erosions at birth over the umbilicus, hands, right ear, and anonychia involving multiple fingers and toes.

At birth, a physical examination revealed erosions at the umbilical base, blistering of the right auricle, multiple tense bullae over the left lower limb, and digital erosions with associated anonychia (Figure 5). An initial mucosal examination revealed intact oral and nasal mucosae and no genital involvement.

**Figure 6:**
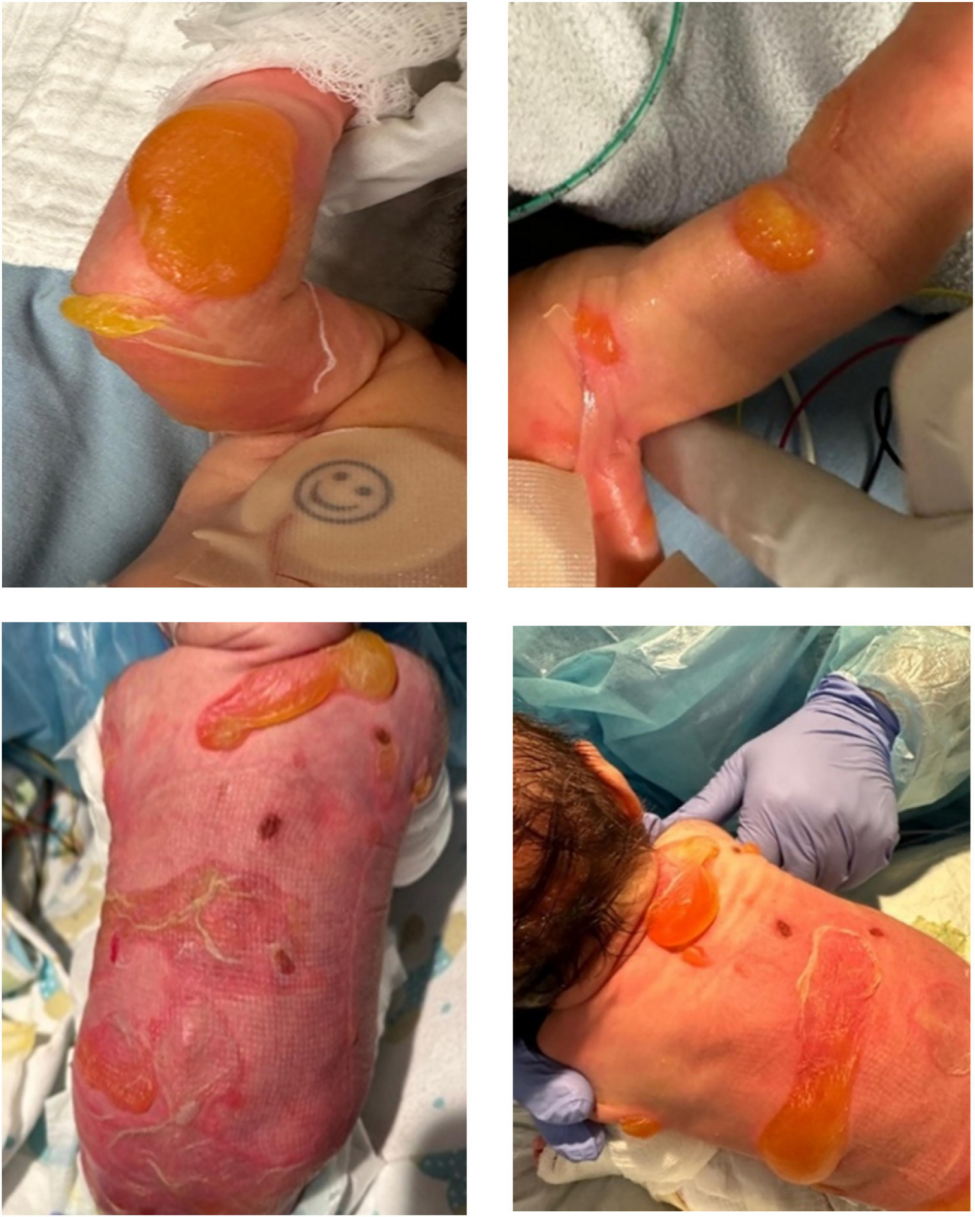
Extensive, large, fluid-filled, and ruptured blisters over the upper extremities and back.

**Figure 7:**
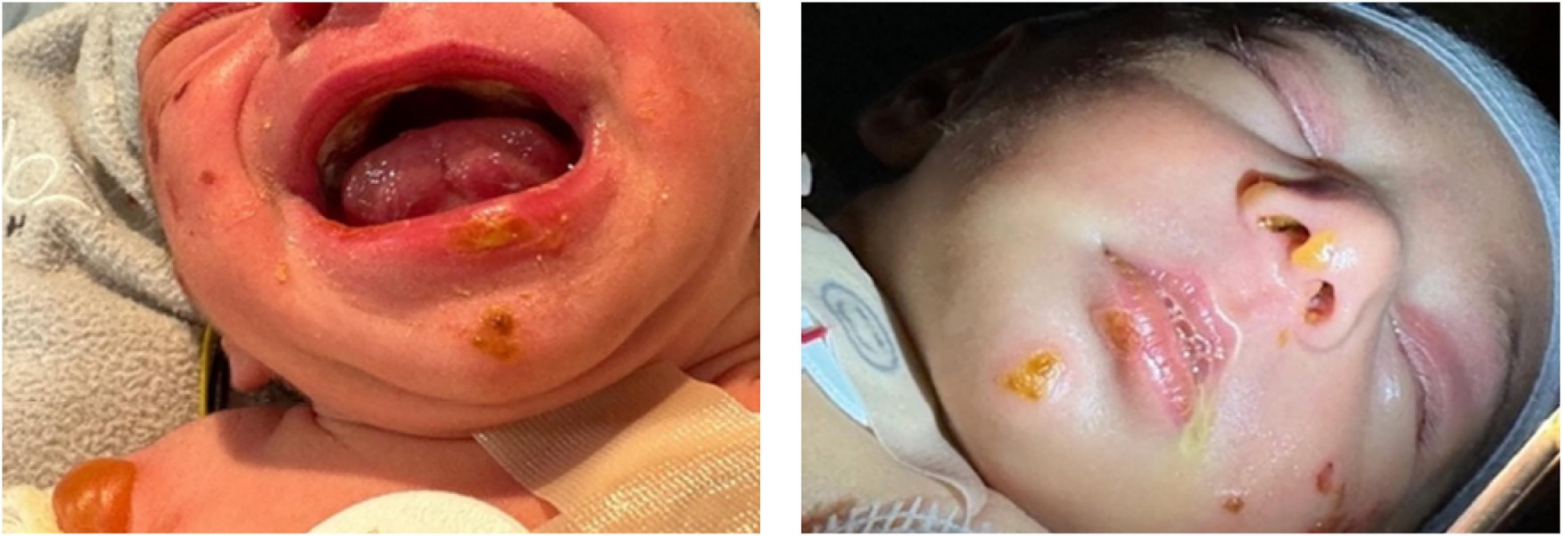
Involvement of the inner mucosa (bullae observed in the nares and soft palate).

The neonate was admitted to the neonatal intensive care unit for monitoring and supportive care. During the first week of life, rapid disease progression occurred, characterized by diffuse tense bullae on the trunk and extremities (Figure 6), progressive nail destruction, and new bullae involving the oral and nasal mucosae (Figure 7). Feeding difficulties developed secondary to mucosal involvement, necessitating nasogastric tube placement.

Echocardiography was performed, and the results were normal, ruling out any associated cardiac abnormalities. Similarly, an abdominal and pelvic ultrasound was performed, showing normal structures and no pyloric or intestinal atresia.

A skin biopsy was not performed because of the lack of diagnostic value. Genetic testing (whole-exome sequencing) was performed for a definitive diagnosis.

Electrolyte disturbances, including hyponatremia and severe hyperkalemia, emerged and were attributed to extensive transepidermal fluid and electrolyte loss. Management included sodium supplementation and nebulized administration of salbutamol. Multimodal analgesia consisted of paracetamol, morphine, and oral ketamine administration. Differential diagnoses included staphylococcal scalded skin syndrome, bullous impetigo, congenital herpes infection, incontinentia pigmenti, and dystrophic EB. Infectious workup was negative. Skin biopsy was deferred because of significant fragility and the availability of molecular diagnostics.

Whole-exome sequencing revealed two heterozygous truncating variants in LAMB3 [NM_000228.3: c.3034C>T, p.(Gln1012*) and c.3247C>T, p.(Gln1083*)], both classified as pathogenic according to ACMG criteria (Class I). These nonsense variants affect exons 20 and 22 and are predicted to result in premature termination and laminin-332 deficiency. In the context of the confirmed trans configuration, the findings are diagnostic of autosomal-recessive junctional epidermolysis bullosa.

These molecular findings confirmed junctional-level cleavage and established a definitive subtype classification. Despite comprehensive supportive management, new blister formation persisted, and the mucosal involvement progressed. At the time of manuscript submission, the infant remained hospitalized with severe disease manifestations, and the long-term prognosis remained guarded.

A structured summary of key clinical events for both cases is presented in Table 1.

**Table 1:** Timeline of clinical events in both neonates.

Day of life (DOL)	Case 1—EB with pyloric atresia	Case 2—Junctional EB (LAMB3 confirmed)
DOL 0 (birth)	Lumbosacral erosions; umbilical and wrist bullae; mucosa intact; NICU admission	Umbilical, ear, and finger erosions; anonychia; leg bullae; NICU admission
DOL 0–1	Abdominal x-ray: single bubble sign suggesting pyloric atresia	Cardiac and abdominal imaging are normal
DOL 1	NPO; total parenteral nutrition started; atraumatic wound care	Supportive wound care initiated
DOL 3–7	Partial healing of initial lesions; limited progression of new bullae	Rapid progression of bullae; worsening nail involvement
DOL 7	Multidisciplinary discussion; parents elected surgical repair	Oral and nasal mucosal bullae; nasogastric feeding initiated
DOL 10	Pyloroplasty performed	Ongoing blister formation; pain management escalated
Postoperative Day 8	Upper gastrointestinal imaging confirmed pyloric patency	–
DOL 18–21	Gradual advancement to full enteral feeds	Electrolyte disturbances (hyponatremia, hyperkalemia) managed; whole-exome sequencing performed
During admission	Genetic testing sent (results pending at submission)	Biallelic pathogenic LAMB3 variants identified
DOL 36	Discharged home with dermatology follow-up	Acute deterioration

## Discussion

Herein, we report two cases of male infants conceived by healthy, non-consanguineous couples originating from different areas of Lebanon, both of whom exhibited pathognomonic features of EB. The worldwide incidence of EB is estimated to be 50 per 1 million live births (21). In light of the scarcity of cases reported worldwide, it was quite peculiar for 2 patients with classic EB features to present to our neonatal intensive care unit within a short time frame.

The first case was associated with whole-exome sequencing. Although a skin biopsy was not performed because of limited diagnostic value in this context, it can aid in assessing severity and inflammation, as well as distinguishing EB from other conditions such as staphylococcal scalded skin syndrome or autoimmune blistering disorders. Antenatal diagnosis was not made in either case, necessitating prompt dermatological and surgical intervention, especially in the first case of EB-PA.

These two cases illustrate the phenotypic heterogeneity of severe neonatal junctional EB associated with distinct molecular defects. Although both involve structural proteins at the dermoepidermal junction, their clinical trajectories differ substantially.

In Case 1, the ITGB4 mutation resulted in EB-PA, characterized by hemidesmosomal dysfunction and gastrointestinal obstruction (3, 22, 23). Historically, EB-PA carries a high mortality rate, despite surgical intervention (21–26). In our patient, the in-hospital postoperative course was comparatively stable, with progressive cutaneous healing and successful establishment of enteral feeding. Nonetheless, long-term prognosis remains uncertain and warrants close follow-up.

The relatively good prognosis in this case highlights the potential variability in EB presentation and underscores the importance of individualized management strategies. Pyloric atresia can be present in different types of EB, but is more frequently associated with the junctional type of EB, identified as Carmi syndrome. The latter has a heterogeneous presentation that carries a high mortality rate despite surgical correction (24). Luo et al. reported the death of two out of four patients in their case series, at the ages of 3 and 4 months. The cause of death was dehydration due to severe gastroenteritis that was resistant to medical treatment (25). In addition, Dank et al. reported that among 51 patients with Carmi syndrome, the average survival rate was 70 days after surgical correction, with sepsis being the cause of mortality (26). The presentation of our patient is remarkable due to the relatively good prognosis, limited progression of skin blistering, and rapid healing of the bullae with no residual scars.

In contrast, the second baby exhibited severe EB without any systemic involvement. The LAMB3 mutation causes laminin-332 deficiency, leading to severe junctional EB. Laminin-332 is essential for anchoring basal keratinocytes to the basement membrane. Severe JEB subtypes are associated with significant neonatal morbidity and mortality (20). The rapid progression of mucocutaneous involvement and electrolyte imbalance observed in this case aligns with previously reported severe phenotypes.

Death was attributed in most cases to septicemia, respiratory failure, electrolyte imbalance, and failure to thrive caused by the severe exudative skin lesions (20, 25). The disease progression in our case was extensive and characterized by the rapid formation of diffuse bullae over the trunk, limbs, fingers, and toes, with subsequent involvement of mucous membranes in various organs and body cavities, scalp, and genitalia. These findings accentuate the challenges in managing severe EB cases and emphasize the critical need for comprehensive, multidisciplinary care to address both cutaneous and systemic manifestations of the disease.

Early molecular confirmation through NGS was pivotal in both cases, allowing precise subtype classification without the need for invasive biopsy (7–14). Accurate genetic diagnosis is fundamental for prognostic assessment, anticipatory management, and genetic counseling.

Novel therapeutic strategies, including gene therapy and stem cell–based approaches, are under investigation (19, 27). Notably, Oleogel-S10, a novel extract gel consisting of 10% birch bark extract in 90% sunflower oil, has shown promising results in accelerating wound healing in patients with inherited dystrophic and junctional EB (27). However, this gel was tested only in infants aged 6 months and older, thus limiting its generalizability. Current treatment remains primarily supportive, emphasizing meticulous wound care, nutritional optimization, and multidisciplinary coordination.

In spite of these advancements, ethical dilemmas persist in such cases regarding treatment escalation in the event of airway compromise, infection, or feeding difficulties, highlighting the need for a collaborative approach involving palliative care specialists, medical professionals, and parents.

The strengths of this report are the molecular confirmation of two distinct severe junctional EB subtypes, detailed neonatal clinical documentation, and demonstration of genotype–phenotype variability within a single tertiary center. Limitations are the absence of immunofluorescence mapping, lack of histopathologic correlation, limited long-term follow-up data, and single-center experience, which may limit generalizability.

## Conclusion

Severe epidermolysis bullosa presenting at birth represents a multisystem disorder requiring prompt recognition and coordinated multidisciplinary management. Gastrointestinal obstruction in neonates with blistering lesions should raise suspicion for EB-PA.

Early molecular diagnosis using next-generation sequencing is critical for accurate classification, prognostic counseling, and family planning. The two cases presented in this study underscore the phenotypic variability of junctional EB associated with ITGB4 and LAMB3 mutations and highlight the essential role of genetic confirmation in guiding neonatal care.

## Data Availability

The original contributions presented in the study are included in the article/Supplementary Material, further inquiries can be directed to the corresponding author.

## References

[1] Bardhan A Bruckner-Tuderman L Chapple ILC Fine JD Harper N Has C Epidermolysis bullosa. Nat Rev Dis Primers. (2020) 6(1):78. 10.1038/s41572-020-0210-0 32973163

[2] Pfendner E Rouan F Uitto J. Progress in epidermolysis bullosa: the phenotypic spectrum of plectin mutations. Exp Dermatol. (2005) 14(4):241–9. 10.1111/j.0906-6705.2005.00324.x 15810881

[3] Gache Y Romero-Graillet C Spadafora A Lépinard C Descamps P Bardon CB A novel homozygous mutation affecting integrin alpha6 in a case of junctional epidermolysis bullosa with pyloric atresia detected in utero by ultrasound examination. J Invest Dermatol. (1998) 111(5):914–6. 10.1046/j.1523-1747.1998.00373.x 9804362

[4] Fine JD Eady RA Bauer EA Briggaman RA Bruckner-Tuderman L Christiano A Revised classification system for inherited epidermolysis bullosa: report of the Second International Consensus Meeting on diagnosis and classification of epidermolysis bullosa. J Am Acad Dermatol. (2000) 42(6):1051–66. 10.1067/mjd.2000.106369 10827412

[5] Wylomanski S Camp G Philippe HJ Le Vaillant C. Épidermolyse bulleuse simplex congénitale de découverte anténatale et apport de l'échographie 3D [Epidermolysis bullosa simplex congenital antenatal discovery and contribution of 3D ultrasound]. Gynecol Obstet Fertil. (2014) 42(6):438–40. [In French.] 10.1016/j.gyobfe.2013.09.004 24411294

[6] Tosto V Herrero B Illescas T De la Calle Fernandez-Miranda M Moreno-Sanz B de Lucas R (New) antenatal ultrasound signs of fetal junctional epidermolysis bullosa: a case report and systematic review of literature. Eur J Obstet Gynecol Reprod Biol. (2023) 290:43–50. 10.1016/j.ejogrb.2023.08.379 37717401

[7] Has C Liu L Bolling MC Charlesworth AV El Hachem M Escámez MJ Clinical practice guidelines for laboratory diagnosis of epidermolysis bullosa. Br J Dermatol. (2020) 182(3):574–92. 10.1111/bjd.18128 31090061 PMC7064925

[8] Mariath LM Kiszewski AE Frantz JA Siebert M Matte U Schuler-Faccini L. Gene panel for the diagnosis of epidermolysis bullosa: proposal for a viable and efficient approach. An Bras Dermatol. (2021) 96(2):155–62. 10.1016/j.abd.2020.05.015 33640189 PMC8007490

[9] Has C Fischer J. Inherited epidermolysis bullosa: new diagnostics and new clinical phenotypes. Exp Dermatol. (2019) 28(10):1146–52. 10.1111/exd.13668 29679399

[10] Takeichi T Liu L Fong K Ozoemena L McMillan JR Salam A Whole-exome sequencing improves mutation detection in a diagnostic epidermolysis bullosa laboratory. Br J Dermatol. (2015) 172(1):94–100. 10.1111/bjd.13190 24947307

[11] Tenedini E Artuso L Bernardis I Artusi V Percesepe A De Rosa L Amplicon-based next-generation sequencing: an effective approach for the molecular diagnosis of epidermolysis bullosa. Br J Dermatol. (2015) 173(3):731–8. 10.1111/bjd.13858 25913354

[12] Has C Küsel J Reimer A Hoffmann J Schauer F Zimmer A The position of targeted next-generation sequencing in epidermolysis Bullosa diagnosis. Acta Derm Venereol. (2018) 98(4):437–40. 10.2340/00015555-2863 29242947

[13] Vahidnezhad H Youssefian L Saeidian AH Touati A Sotoudeh S Abiri M Multigene next-generation sequencing panel identifies pathogenic variants in patients with unknown subtype of epidermolysis Bullosa: subclassification with prognostic implications. J Invest Dermatol. (2017) 137(12):2649–52. 10.1016/j.jid.2017.07.830 28830826

[14] Lucky AW Dagaonkar N Lammers K Husami A Kissell D Zhang K. A comprehensive next- generation sequencing assay for the diagnosis of epidermolysis bullosa. Pediatr Dermatol. (2018) 35(2):188–97. 10.1111/pde.13392 29334134

[15] Marinkovich MP Tang JY. Gene therapy for epidermolysis Bullosa. J Invest Dermatol. (2019) 139(6):1221–6. 10.1016/j.jid.2018.11.036 31068252

[16] Hirsch T Rothoeft T Teig N Bauer JW Pellegrini G De Rosa L Regeneration of the entire human epidermis using transgenic stem cells. Nature. (2017) 551(7680):327–32. 10.1038/nature24487 29144448 PMC6283270

[17] Mavilio F Pellegrini G Ferrari S Di Nunzio F Di Iorio E Recchia A Correction of junctional epidermolysis bullosa by transplantation of genetically modified epidermal stem cells. Nat Med. (2006) 12(12):1397–402. 10.1038/nm1504 17115047

[18] Melo SP Lisowski L Bashkirova E Zhen HH Chu K Keene DR Somatic correction of junctional epidermolysis bullosa by a highly recombinogenic AAV variant. Mol Ther. (2014) 22(4):725–33. 10.1038/mt.2013.290 24390279 PMC3982486

[19] Latella MC Cocchiarella F De Rosa L Turchiano G Gonçalves MAFV Larcher F Correction of recessive dystrophic epidermolysis Bullosa by transposon-mediated integration of COL7A1 in transplantable patient-derived primary keratinocytes. J Invest Dermatol. (2017) 137(4):836–44. 10.1016/j.jid.2016.11.038 28027893

[20] Fine JD Johnson LB Weiner M Suchindran C. Cause-specific risks of childhood death in inherited epidermolysis bullosa. J Pediatr. (2008) 152(2):276–80. 10.1016/j.jpeds.2007.06.039 18206702

[21] Luo C Yang L Huang Z Su Y Lu Y Zhang M Case report: a case of epidermolysis bullosa complicated with pyloric atresia and a literature review. Front Pediatr. (2023) 11:1098273. 10.3389/fped.2023.1098273 37033187 PMC10076629

[22] Chung HJ Uitto J. Epidermolysis bullosa with pyloric atresia. Dermatol Clin. (2010) 28(1):43–54. 10.1016/j.det.2009.10.005 19945615 PMC2790914

[23] Lucky AW Gorell E. Epidermolysis bullosa with pyloric atresia. In: Adam MP Feldman J Mirzaa GM, editors. GeneReviews®. Seattle (WA): University of Washington, Seattle (2008). p. 1993–2024. 20301336

[24] Al Faqeeh AA Syed MK Hussain S Almas T Ammar M. Carmi syndrome in a neonate: an exacting surgical challenge. Cureus. (2020) 12(9):e10522. 10.7759/cureus.10522 33094063 PMC7574818

[25] Chahed J Mekki M Ksia A Kechiche N Hidouri S Youssef TM Management of digestive lesions associated to congenital epidermolysis bullosa. Afr J Paediatr Surg. (2015) 12(4):221–6. 10.4103/0189-6725.172544 26712284 PMC4955475

[26] Dank JP Kim S Parisi MA Brown T Smith LT Waldhausen J Outcome after surgical repair of junctional epidermolysis bullosa–pyloric atresia syndrome: a report of 3 cases and review of the literature. Arch Dermatol. (1999) 135(10):1243–7. 10.1001/archderm.135.10.1243 10522673

[27] Heo YA. Birch bark extract: a review in epidermolysis Bullosa. Drugs. (2023) 83(14):1309–14. 10.1007/s40265-023-01935-z 37658982

